# Conduction Threshold in Accumulation-Mode InGaZnO Thin Film Transistors

**DOI:** 10.1038/srep22567

**Published:** 2016-03-02

**Authors:** Sungsik Lee, Arokia Nathan

**Affiliations:** 1 Electrical Engineering Division, Department of Engineering, University of Cambridge 9 JJ Thomson Avenue, Cambridge CB3 0FA, United Kingdom

## Abstract

The onset of inversion in the metal-oxide-semiconductor field-effect transistor (MOSFET) takes place when the surface potential is approximately twice the bulk potential. In contrast, the conduction threshold in accumulation mode transistors, such as the oxide thin film transistor (TFT), has remained ambiguous in view of the complex density of states distribution in the mobility gap. This paper quantitatively describes the conduction threshold of accumulation-mode InGaZnO TFTs as the transition of the Fermi level from deep to tail states, which can be defined as the juxtaposition of linear and exponential dependencies of the accumulated carrier density on energy. Indeed, this permits direct extraction and visualization of the threshold voltage in terms of the second derivative of the drain current with respect to gate voltage.

Thin film transistors (TFTs) with disordered channel layers comprising say amorphous oxide or organic semiconductors, are becoming ubiquitous especially where low temperature processability and flexibility are essential^1^,^2^,^3^. To facilitate TFT systems design, a complete understanding on device physics and related device parameter extraction constitutes the first and inevitable step. In this vein, the work presented here on conduction threshold is a very fundamental device parameter. The work is applicable not only to thin film field effect transistor (FET) but also the pervasive silicon metal-oxide-semiconductor FET (MOSFET). Given its significance as a crucial indicator of carrier transport and instability in FETs^4^,^5^,^6^,^7^,^8^,^9^,^10^, the conduction threshold has to be determined physically rather than by empirical means^11^.

Silicon MOSFETs conduct in inversion mode and the physical reason underlying the conduction threshold is well understood^4^,^5^. It corresponds to the critical state in which the induced or (inverted) carrier density becomes the same as the substrate’s majority carrier density^5^. For example, in an n-channel MOSFET, this transition point is when the electron density goes from linear to exponential dependence on energy in the solution of the Poisson-Boltzmann equation 5. This transition can be approximated empirically as the intercept made from a linear extrapolation on the drain current (I_DS_) vs. gate voltage (V_GS_) characteristic at very low drain voltage (V_DS_)^5^.

In contrast to the silicon MOSFET, the TFT works in carrier accumulation mode regardless of the channel material^6^,^7^,^8^,^9^. This means that there is no polarity inversion of the induced carriers, so it is difficult to physically define the accumulation threshold. Moreover, because of the structural disorder of the semiconductor channel layer, there are localized deep and tail states distributed within the so-called band-gap, and depending on the position of the Fermi-level, free carriers or trapped carriers can be prevalent^12^,^13^. As a result, the conduction threshold is difficult to extract even empirically^6^,^14^,^15^,^16^. The density of carriers trapped at these localized states can be greater than free carriers, and there is no physical criteria for which all the traps are filled. In this family of devices, it is the localized trap states that determine the behaviour of field-effect mobility as a function of gate bias.

In this letter, we present a systematic analysis of the conduction threshold leading to a physical definition of the threshold voltage in accumulation-mode InGaZnO TFTs. By solving the Poisson-Boltzmann equation coupled with measurements of the current-voltage characteristics, the total carrier density is captured as a function of the surface potential. Similar to the MOSFET, it turns out that there is a transition point in which the accumulated carrier concentration switches from a linear to an exponential dependence on energy, i.e. the free carrier density increases rapidly from this threshold level by virtue of trap-limited conduction. We find that this threshold can be directly extracted as the gate voltage in which the second derivative of I_DS_ with respect to V_GS_ peaks. This is visualized with the proposed grayscale image spectroscopy, allowing an ease of analysis on threshold voltage and related properties. The studies presented here show that the conduction threshold is independent of the band mobility and is largely determined by the sub-threshold characteristics associated with the deep states including interface states.

## Results and Discussion

### Microscopic conduction threshold

**Figure 1:**
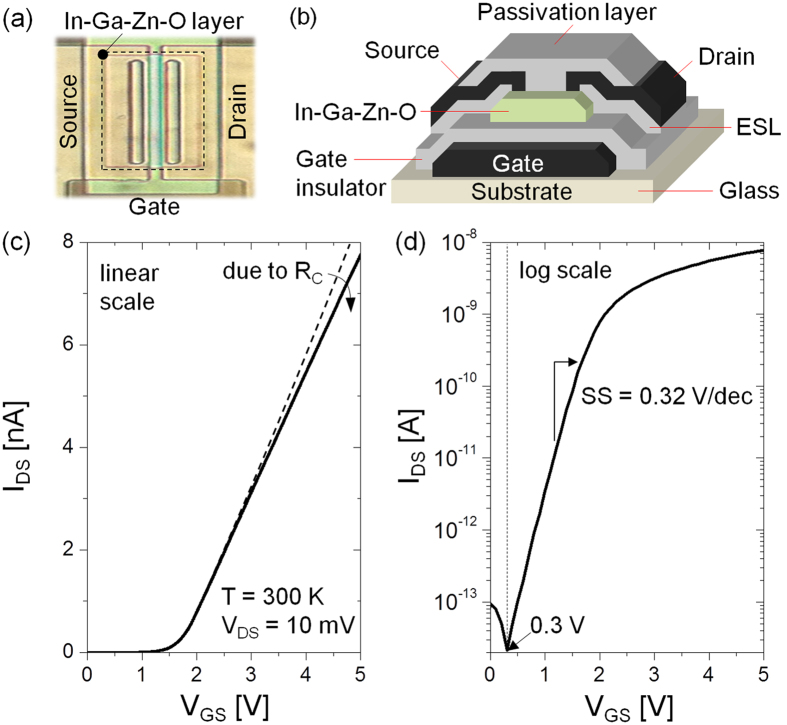
(**a**) Photomicrograph and (**b**) schematic of the fabricated InGaZnO TFTs examined in this work. Measured drain current (I_DS_) vs. gate voltage (V_GS_) in (**c**) linear scale and (**d**) log scale, respectively. Here, we indicate an effective flat-band voltage ~0.3 V.

**Figure 2:**
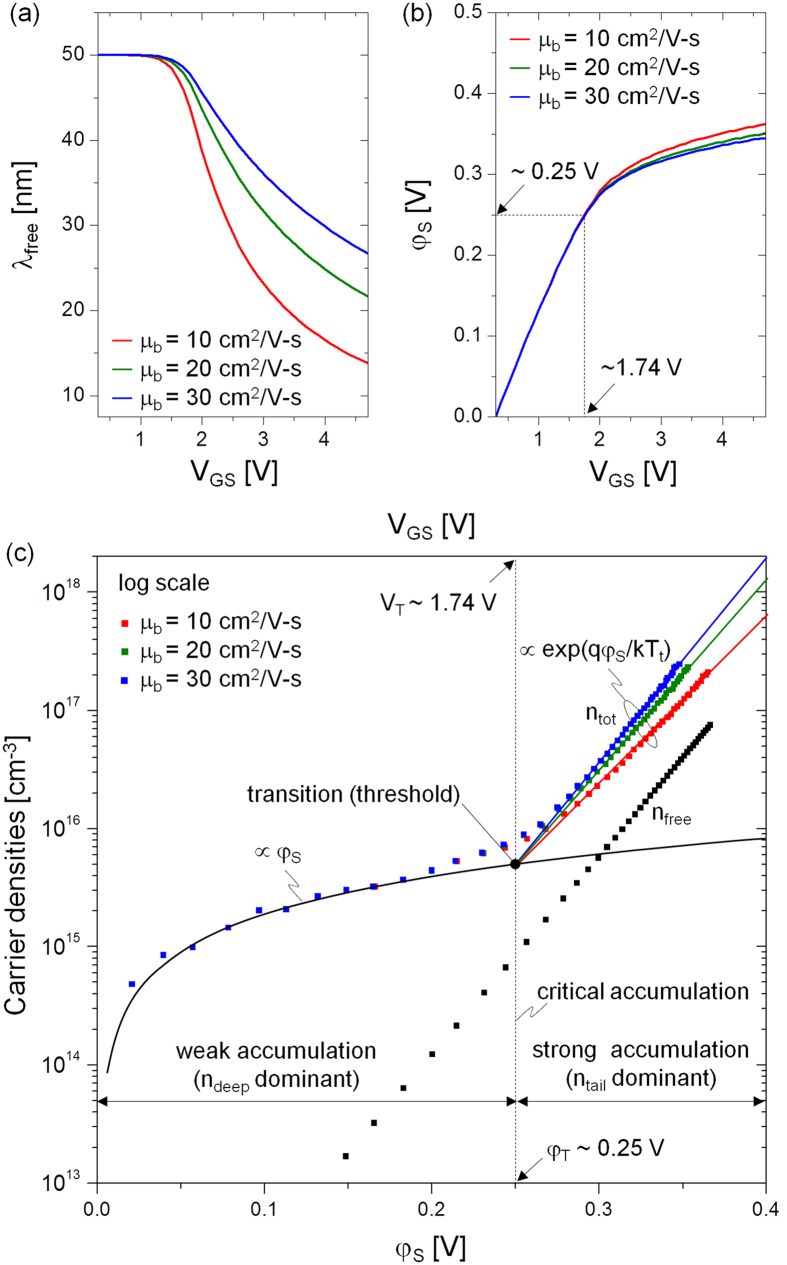
(**a**) Effective thickness of induced free carrier sheet (λ_free_) vs. V_GS_. (**b**) Surface potential (φ_S_) as a function of V_GS_ and (**c**) total induced carrier density (n_tot_) and n_free_ as a function of φ_S_ for different μ_b_ values of 10, 20, and 30 cm^2^/V-s, respectively.

For the investigations reported here, we used a semiconducting oxide TFT test structure with a 50 nm thick InGaZnO channel layer, as seen in Fig. 1(a,b). The device is a typical bottom gate structure where Mo is used for electrodes. Here, the gate insulator is formed as a bilayer of SiO_x_ and SiN_x_, and the total gate-insulator capacitance of this structure (C_ox_) is ~10 nF/cm^2^. As a final step, a single layer of SiO_x_ is used for passivation layer and etch-stop layer (ESL). The TFT has a 50 μm channel width (W) and 10 μm channel length (L). Its I_DS_ vs. V_GS_ measured for V_DS_ fixed at 10 mV is shown in Fig. 1(c,d). Here, we retrieve an effective flat-band voltage (V_FB_) of 0.3 V and a sub-threshold slope (SS) of 0.32 V/dec. Using the measured I_DS_ − V_GS_ characteristics, the microscopic picture in terms of free and trapped carrier densities can be captured as a function of V_GS_. The free charge density (Q_free_) relates to the drain current as^12^,^13^,


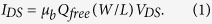


where μ_b_ is the band mobility. Equation (1) is valid for both sub- and above-threshold regimes as long as V_DS_ < kT/q, where kT thermal energy and q the elementary charge (see Supplementary Information S1). In addition, we replace V_DS_ with V_DS_ − R_C_I_DS_, where R_C_ is a contact resistance. The value of R_C_ retrieved for the test structure considered here is 50 kΩ. The main-unknown in Equation (1) is μ_b_, which typically ranges from 10 to 30 cm^2^/V-s^7^,^9^. From Equation(1) and the measured I_DS_ − V_GS_, Q_free_ can be stated as a function of V_GS_, and related with the free carrier density (n_free_) through Q_free_ = q n_free_λ_free_. Here, λ_free_ is an effective thickness of the induced free carrier sheet, and is expressed as an harmonic average between the free carrier Debye length (λ_D_) and thickness of In-Ga-Zn-O channel layer (t_S_), viz. λ_free_ = (λ_D_^−1^ + t_S_^−1^)^−1^, assuming that the penetration depth of the vertical electric field is limited within t_S_^17^,^18^. The λ_D_ can be obtained using ε_S_kT/(qQ_free_)^5^,^16^, where ε_S_ is the permittivity of the In-Ga-Zn-O channel layer. The value of λ_free_ is shown in Fig. 2(a). From this, we express n_free_ as Q_free_/(qλ_free_), which is still a function of V_GS_, i.e. n_free_(V_GS_). To express it as a function of surface potential (φ_S_), we relate this to Boltzmann’s equation, i.e. n_free_ = N_C_ exp[(E_F0_ + qφ_S_ – E_C_)/kT]^5^,^12^. Here, N_C_ is the effective density of free carriers, E_F0_ an equilibrium Fermi level, and E_C_ the energy level of the conduction band minima. For the device considered here, N_C_ is ~2 × 10^18 ^cm^−3^. We now have a correspondence between V_GS_ and φ_S_,


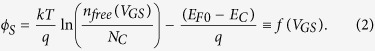


Using Equation (2), φS is computed for three different values of μ_b_, which is contained in n_free_(V_GS_). This is shown in Fig. 2(b).

Now, we solve the integral form of Poisson’s equation, which yields the relationship between the surface electric field (E_S_) and integral of total accumulated carrier density n_tot_ for φ_S_ as follows^12^,


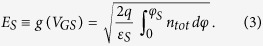


For more detailed derivation, see Supplementary Information S2. In addition, we have expressed the left hand side of the Equation (3) as a function of V_GS_, i.e. E_S_ = C_ox_(V_GS_ − V_FB_)/ε_S_ ≡ g(V_GS_) by virtue of Gauss’ Law (see Supplementary Information S3). Based on Equations (2) and (3), n_tot_ can be written as,


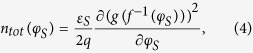


in which n_tot_ is n_free_ + n_deep_ + n_tail_. Here n_deep_ and n_tail_ are the densities of carriers trapped in deep and tail states, respectively. In the term, n_deep_, the effect of carriers trapped at the interface states is embedded since n_deep_ consists of defect state carriers at the bulk (i.e. n_bulk_) as well as interface (i.e. n_int_), thus n_deep_ = n_bulk_ + n_int_^13^,^17^. Equation (4) can be considered as a differential form of the Poisson-Boltzmann equation, allowing examination of the behaviour of carrier densities n_tot_ with φ_S_, as depicted in Fig. 2(c) in a semi-log plot. A transition is observed at φ_S_ = 0.25 V, for a corresponding gate voltage of 1.74 V, whereby the carrier density n_tot_ goes from linear to an exponential dependency on energy. Based on this observation, we can define the threshold voltage (V_T_) of the device as 1.74 V, which corresponds to the transition surface potential (φ_T_) of 0.25 V. The physical meaning underlying this transition is further discussed in the following.

From Fig. 2(c), we find that the trapped carriers are dominant for all energy levels, i.e. n_free_ < n_tail_ + n_deep_, thus trap-limited conduction (TLC) always prevails. Depending on the value of φ_S_, the one or the other can prevail. For example, below φ_T_, n_deep_ prevails and the linear dependency of n_tot_ can be explained as,


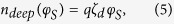


where ζ_d_ is a deep state density related to either n_bulk_ or n_int_. From Fig. 2(c), the value of ζ_d_ in the examined InGaZnO TFT is estimated as ~2.1 × 10^16 ^cm^−3^ eV^−1^. Besides, ζ_d_ can also be retrieved from the relation C_deep_/(q^2^t_S_), where C_deep_ is a deep state capacitance, which can be deduced independently from the sub-threshold slope (SS) through the relationship, C_deep_ = C_ox_ [SS/(kT/q·ln10) – 1]^17^. For the channel thickness considered here (t_S_ = 50 nm), C_deep_ ~ 43.7 nF/cm^2^, yielding ζ_d_ ~ 3 × 10^16 ^cm^−3^ eV^−1^. It is consistent with the value empirically estimated using Fig. 2(c). Note that, along with the C_deep_ and φ_T_, an analytical expression of threshold voltage (V_T_) can be derived from the charge neutrality equation, C_ox_(V_T_ − V_FB_ − φ_T_) = C_deep_φ_T_, yielding as,


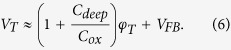


With Equation (6), V_T_ is estimated as 1.64 V using C_deep_ ~ 43.7 nF/cm^2^, C_ox_ ~ 10 nF/cm^2^, φ_T_ = 0.25 V and V_FB_ = 0.3 V. Although it may be inaccurate due to its approximation to be analytical, Equation (6) can allow a quick estimation of V_T_. Also, it is useful to see its proportionality with deep states (i.e. bulk and interface states) through the term, C_deep_, for example.

After the transition (φ_T_ = 0.25 V), n_tail_ prevails. Indeed, the carrier density now increases exponentially, seen as linear on the semi-log plot Fig. 2(c). And it can be described by the following relation for kT_t_ > kT^12^,^16^,^19^,


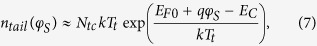


where N_tc_ is the tail state density at E_C_ and kT_t_ is the characteristic energy of tail states associated with the exponential profile of the energy of the band tail states^6^,^12^,^14^,^16^,^19^. As seen in Fig. 2(c), the transition is always maintained as an intersection point regardless of the assumed value of μ_b_.

To check the relationship between the transition point and electronic states for electrons, we deduce the density of states using the values of n_tot_ and n_free_ (see Fig. 2(c)), through^12^,


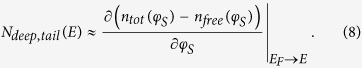


**Figure 3:**
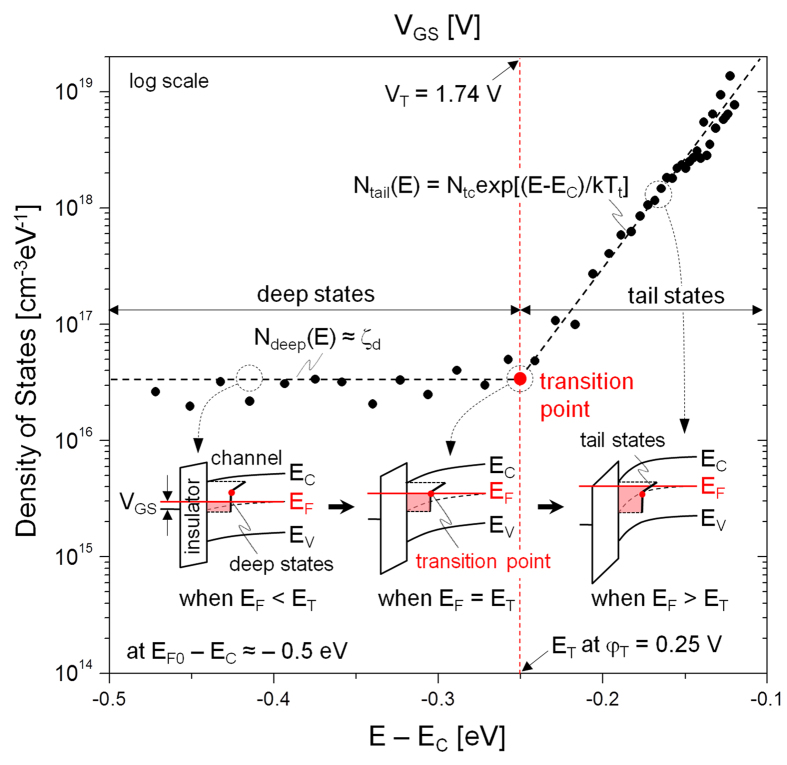
Density of states as a function of energy from E_C_. Inset: band diagrams for three different cases of transition energy (E_T_): below E_T_, at E_T_, and above E_T_. Here, E_V_ denotes the valence band maxima, and N_deep_(E) and N_tail_(E) the respective density of deep and tail states as a function of energy.

Here, the Fermi level E_F_ replaces φ_S_ by virtue of the relation, E_F_ − E_F0_ = qφ_S_. Figure 3 shows the profile of the density of states as a function of energy while assuming μ_b_ = 20 cm^2^/V-s. We identify a transition energy E_T_ that demarcates the deep and tail states, confirming that all the deep states (N_deep_) are filled when E_F_ arrives at E_T_ (see also the inset of Fig. 3). This demarcation defines the threshold voltage. Based on this observation of E_T_, it is expected that E_T_ can move toward a higher energy when N_deep_ is increased while maintaining the profile of tail states, resulting in a higher V_T_ (see Supplementary Information S6). As seen in Fig. 3, at energies higher than E_T_, the tail state distribution is found to follow an exponential dependence, i.e. N_tail_(E) = N_tc_exp[(E − E_C_)/kT_t_]^6^,^12^,^16^. Using the exponential dependence of the tail states, the retrieved values of N_tc_ and kT_t_ are ~10^20 ^cm^−3^ eV^−1^ and 27 meV, respectively, as seen in Fig. 3. Thus kT_t_ > kT at T = 300 K. This confirms that the TLC is dominant at T = 300 K. Besides, the exponent (α) in the power-law for the I_DS_ − V_GS_ curve above V_T_ is defined as 2 kT_t_/kT – 1 for kT_t_ > kT^6^,^12^,^14^,^20^. The value of α at T = 300 K is about 1.07 for kT_t_ = 27 meV, which consistent with the observed linear dependence of I_DS_ on V_GS_ as seen in Fig. 1(c). As implied here, the role of N_tc_ and kT_t_ is important in the TFT conduction, relating to the material composition. For example, with decreasing the ratio of In/Ga, field effect mobility of In-Ga-Zn-O TFTs can be reduced, suggesting a higher density of tail states (N_tc_)^21^,^22^. In other words, if N_tc_ is higher, E_F_ can be pinned at a lower energy from the conduction band minima (see Supplementary Information S6). This leads to a lower mobility reminiscent of the a-Si TFT whereas Fermi level in oxide TFT can even go into the conduction band due to its lower tail state density^23^.

Based on the characteristic behaviour of the carrier densities shown in Fig. 2(c), the significance of the free carrier density (n_free_) relative to the total carrier density (n_tot_ = n_free_ + n_deep_ + n_tail_) can be defined as a ratio (χ_TLC_) in accordance to the TLC theory^6^,^14^,^16^,


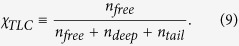


**Figure 4:**
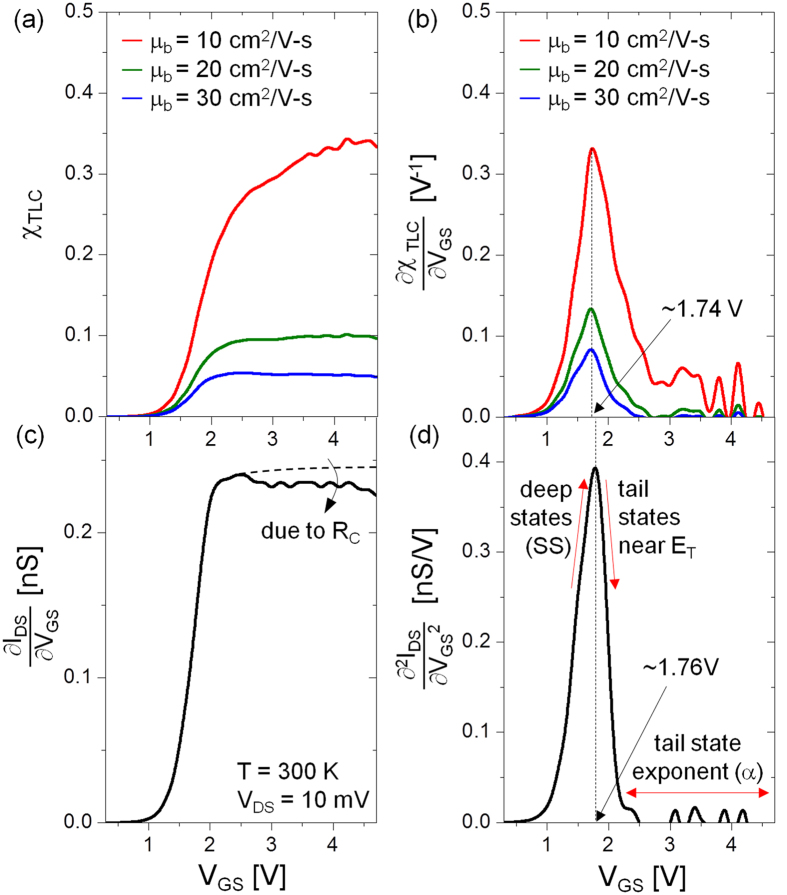
(**a**) Trap-limited conduction (TLC) ratio (χ_TLC_) as a function of V_GS_. (**b**) The first derivative of χ_TLC_ with respect to V_GS_. (**c**) The first derivative (i.e. ∂I_DS_/∂V_GS_) and (**d**) second derivative (i.e. ∂^2^I_DS_/∂V_GS_^2^) of I_DS_ vs. V_GS_.

Using Equation (8) together with the values shown in Fig. 2(c), the behaviour of χ_TLC_ and its first derivative with respect to V_GS_ (i.e. ∂χ_TLC_/∂V_GS_) as a function of V_GS_ is shown in Fig. 4(a,b), respectively. We see that ∂χ_TLC_/∂V_GS_ peaks at V_GS_ ~ 1.74 V regardless of the value of μ_b_. This is consistent with our earlier observation of the common intercept seen in Fig. 2(c). Indeed, χ_TLC_ shows a similarity with the first derivative of the current-voltage characteristics, ∂I_DS_/∂V_GS_, as seen in Fig. 4(a,c). As it turns out, ∂I_DS_/∂V_GS_ is the trans- conductance of the transistor, which in turn is proportional to the field effect mobility (μ_FE_). Thus, μ_FE_ is proportional to χ_TLC_. Indeed in accordance with the TLC theory, μ_FE_ can be defined as μ_b_ n_free_/n_tot_ = μ_b_ χ_TLC_^6^,^20^,^24^. At high V_GS_, the behaviour of ∂I_DS_/∂V_GS_ without effect of R_C_ looks similar to χ_TLC_ for μ_b_ = 20 cm^2^/V-s (see Supplementary Information S4). This suggests that μ_b_ of the examined device is ~20 cm^2^/V-s. The similarity between ∂χ_TLC_/∂V_GS_ and the second derivative ∂^2^I_DS_/∂V_GS_^2^ becomes even more striking in which the peak is at 1.74 V regardless of the band mobility (μ_b_) values assumed for this analysis (see Fig. 4(b)). More importantly, the experimentally observed peak in ∂^2^I_DS_/∂V_GS_^2^ vs. V_GS_ appears at ~1.76 V which corroborates with theoretical prediction with a discrepancy less than 2%, as seen in Fig. 4(d). From the nature of the ∂^2^I_DS_/∂V_GS_^2^ curve, we clearly identify the roles of deep and tail states, respectively, on either side of the peak where the threshold voltage lies. The deep states and corresponding sub-threshold slope determines the slope of the rising edge while the tail states and its exponent α are associated with that of the falling edge (see Fig. 4(d)).

### Macroscopic observation of the threshold

**Figure 5:**
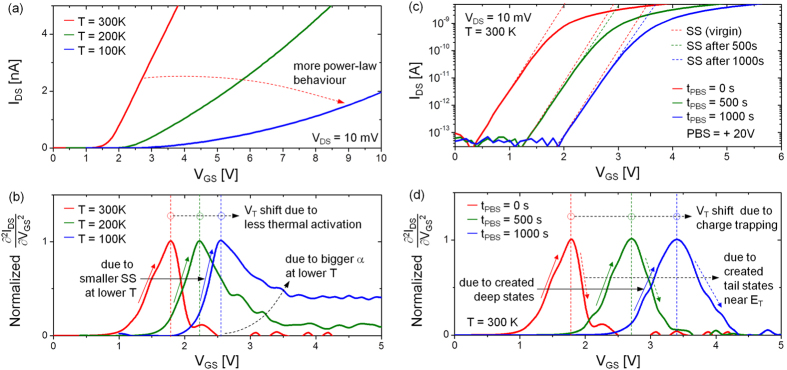
(**a**) Measured I_DS_ vs. V_GS_ for three different temperatures (T = 300 K, 200 K, and 100 K). (**b**) Normalized ∂^2^I_DS_/∂V_GS_^2^ for different temperatures. (**c**) Three curves of I_DS_ vs. V_GS_ measured before and after +20 V positive bias stress (PBS) for 500s and 1000s, respectively. (**d**) Normalized ∂^2^I_DS_/∂V_GS_^2^ for different bias stress times. Note that each ∂^2^I_DS_/∂V_GS_^2^ has been normalized to its peak value.

The above analysis can be extended to measurements of device behaviour at different temperatures. Figure 5(a) shows I_DS_ − V_GS_ characteristics at T = 300 K, 200 K, 100 K, and the ∂^2^I_DS_/∂V_GS_^2^ in each case is shown in Fig. 5(b). As the ambient temperature decreases, we observe that V_T_ shifts to more positive V_GS_ values. This is because of reduced thermal activation^12^. As temperature decreases, the current-voltage characteristic approaches a more power-law behaviour, as indicated in Fig. 5(a). Consequently, its second derivative converges to a non-zero value for V_GS_ > V_T_, and which increases with decreasing temperature, as seen in Fig. 5(b). This is mainly associated with the tail state exponent (i.e. α = 2 kT_t_/kT–1), which increases with decreasing temperature. In contrast, the rising edge becomes increasingly steeper at lower temperatures, as indicated in Fig. 5(b). This is mainly due to the smaller sub-threshold slope (SS) at lower temperatures, which is typical of field effect transistors and can be intuitively explained with the relation, SS = ln(10)·kT/q(1 + C_deep_/C_ox_)^17^, assuming C_deep_ is independent of temperature.

The observations of the current-voltage characteristics and its second derivative presented hitherto considerably simplify the interpretation of the shift in threshold voltage in bias stress experiments. More importantly, the shift in V_T_ can be retrieved with minimizing an interruption to the stress measurement conditions. Figure 5(c) shows the measured I_DS_ − V_GS_ characteristics following positive bias stress (i.e. PBS at V_GS_ = +20 V while maintaining V_DS_ = 0 V) for stress durations (t_PBS_) of 500 sec and 1000 sec. As seen in Fig. 5(d), there is a positive V_T_-shift, which increases with stress duration. Since the value of ∂^2^I_DS_/∂V_GS_^2^ in a well-above V_T_ regime remains zero for all cases, as seen in Fig. 5(d), it suggests that there is no significant degradation in band tail states (e.g. kT_t_), thus unaffecting the tail state exponent α. This suggests electron trapping into the gate insulator as the significant and likely source of V_T_-shift. However, the ∂^2^I_DS_/∂V_GS_^2^ curve appears to spread out more for longer t_PBS_. Indeed, the slope of the rising edge (at V_GS_ < V_T_) becomes less steep, suggesting creation of deep defects^25^,^26^,^27^. Since the slope of falling edge decreases, as seen in Fig. 5(d), it can be argued that tail states are newly created near E_T_ (see Supplementary Information S5). So, it is obvious that the analysis presented here is especially useful under bias stress, as it is indicative of the nature of defects created in the sub-threshold and above-threshold regimes.

**Figure 6:**
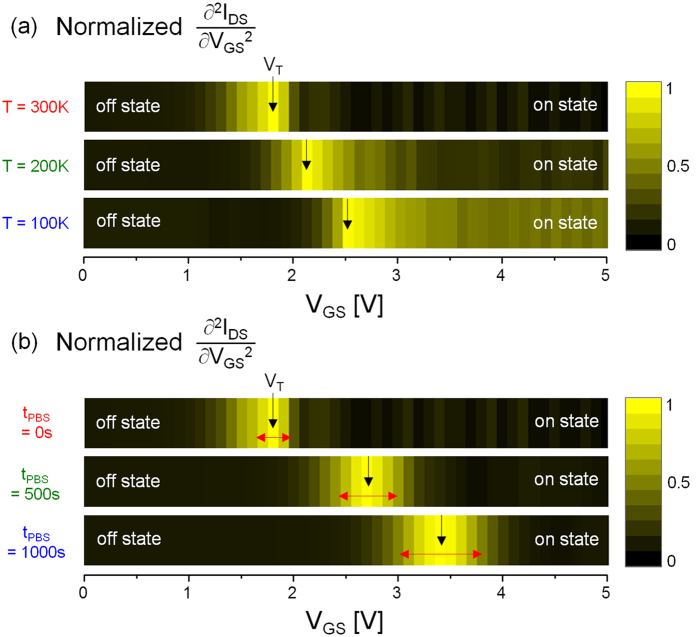
Grayscale images constructed from ∂^2^I_DS_/∂V_GS_^2^ (**a**) for different temperatures, and (**b**) for different stress durations, respectively.

As another outcome of the presented analysis, we propose an image spectroscopy constructed from the second derivatives, as seen in Fig. 5. This method allows a visualization of V_T_ position as well as a property change near V_T_. As an example, Fig. 5(a) shows I_DS_ − V_GS_ characteristics measured at different ambient temperatures (300 K, 200 K, 100 K), and the V_T_ for each case is visualized in a grayscale image, as indicated in Fig. 6(a). Here, it is found that V_T_ is shifted to more positive in V_GS_ as decreasing temperature. Besides this, it is also observed that the grayscale image above V_T_ is getting brighter as decreasing temperature. This confirms that I_DS_ − V_GS_ curvature in on state (i.e. above-threshold regime) becomes more nonlinear, as can be seen in Fig. 5(b). As another example, it can be applied to analyze the V_T_ instability with respect to bias stresses. In Fig. 5(c), we show the measured I_DS_ − V_GS_ curves after applying a positive bias stress (V_GS_ = +20 V while V_DS_ = 0 V) for 500 sec and 1000 sec, respectively. As clearly seen in the constructed grayscale image (see Fig. 6(b)), there is a positive V_T_ shift and its increase towards more positive direction in V_GS_ with a longer stress time (t_PBS_). Since the on state image brightness for each case looks dark and similar each other, as seen in Fig. 6(b), we can say that there isn’t a significant degradation in band tail states which represents above-threshold regime (i.e. on state) curvature. So, this can be explained with electron trapping into gate insulator. However, the bright parts near V_T_ appear to spread out more after applying the PBS with a longer t_PBS_. This suggests that the sub-threshold slope has been slightly bigger, and tail states are newly created near E_T_. This is consistent with the results in Fig. 5(d).

## Conclusions

The channel layer in amorphous thin film transistors in accumulation mode generally suffer from structural disorder resulting in localized deep and tail states in the energy gap. This makes the classical empirically-based extraction of the conduction threshold difficult, if not ambiguous, since depending on the Fermi level, conduction of free or trapped carriers can be prevalent determined by the shape of the localized tail states. This paper resolves the ambiguity in interpretation of the conduction threshold in accumulation-mode InGaZnO thin film transistors and provides a quantitative means of uniquely extracting the threshold voltage. The conduction threshold has been identified to take place at the transition of the Fermi level from deep to tail states, following which the carrier density goes from a linear to exponential dependence on energy. While the analysis presented here can be applied to study the relative changes in deep defects and tail states at pre- and post-transition caused by effects of ambient temperature and bias stress, a more precise analytical description is needed to quantitatively assess and subsequently predict the nature of the density of states when subject to environmental factors. Work along these lines is in progress. Nevertheless the results demonstrated here give physical and quantitative insight into the V_T_ and related properties in accumulation-mode thin film transistors.

## Additional Information

**How to cite this article**: Lee, S. and Nathan, A. Conduction Threshold in Accumulation-Mode InGaZnO Thin Film Transistors. *Sci. Rep.* **6**, 22567; doi: 10.1038/srep22567 (2016).

## Supplementary Material

Supplementary Information
