# Supplementary material for: Conduction Threshold in Accumulation-Mode InGaZnO Thin Film Transistors
Source: Sci Rep. 2016 Mar 2;6:22567. doi: 10.1038/srep22567 (PMC4773861; doi:10.1038/srep22567)
Supplement: Supplementary Information [file srep22567-s1.doc]

**Supplementary Information**

**Conduction Threshold in Accumulation-Mode InGaZnO Thin Film Transistors**

**Sungsik Lee^1^ and Arokia Nathan^2^**

Electrical Engineering Division, Department of Engineering, University of Cambridge,

9 JJ Thomson Avenue, Cambridge CB3 0FA, United Kingdom

(Emails: ^1^ [sl684@cam.ac.uk](mailto:sl684@cam.ac.uk), ^2^ [an299@cam.ac.uk](mailto:an299@cam.ac.uk))

**█ S1. Relationship between free charge (Q_free_) and drain current (I_DS_)**

For sub-threshold regime, I_DS_ is derived from diffusion current definition [S1-1], as follows,

$I_{DS}=\mu_{b}\frac{W}{L}\frac{kT}{q}Q_{free}\left(1-\exp\left(-\frac{qV_{DS}}{kT}\right)\right)$, (S1-1)

$Q_{free}=Q_{0}\exp\left(\frac{q(V_{GS}-V_{FB})}{kT\left(1+C_{deep}^{*}/C_{ox}\right)}\right)$. (S1-2)

Here, the assumption, V_DS_<kT/q, yields the following formula through the Taylor expansion,

$1-\exp\left(-\frac{qV_{DS}}{kT}\right)=1-\left(1-\frac{qV_{DS}}{kT}+higher order terms\right)\approx\frac{qV_{DS}}{kT}$, (S1-3)

With Eq.(S1-3), Eq.(S1-1) is now simplified as follows,

$I_{DS}(sub-threshold)=\mu_{b}Q_{free}\frac{W}{L}V_{DS}$. (S1-4)

For above-threshold regime, I_DS_ is given from drift current definition [S1-1], as follows,

$I_{DS}=\mu_{FET}\frac{W}{L}Q_{tot}V_{DS}$. (S1-5)

Here, the product of Q_tot_ and μ_FET_ can be transformed into its equivalence as follows [S1-2],

$\mu_{FET}Q_{tot}=\mu_{b}Q_{free}$. (S1-6)

With Eq.(S1-6), Eq.(S1-5) is now rewritten in terms of Q_free_,

$I_{DS}(above-threshold)=\mu_{b}Q_{free}\frac{W}{L}V_{DS}$. (S1-7)

Hence, as seen in Eqs.(S1-4) and (S1-7), both regimes current can be unified as,

$I_{DS}=\mu_{b}Q_{free}\frac{W}{L}V_{DS}$. (S1-8)

**References:**

[S1-1] S. Lee *et al.,* *J. Display Tech*. **9**(11), 883 (2013).

[S1-2] P. Servati et al., *IEEE Tran. Elec. Devi*. **50**(11), 2227 (2003).

**█ S2. Derivation of the Poisson-Boltzmann Equation in a differential form**

Firstly, a 2-dimensional Poisson’s equation is represented as follows,

$\frac{\partial^{2}\phi(x,y)}{\partial x^{2}}+\frac{\partial^{2}\phi(x,y)}{\partial y^{2}}=\frac{q}{\varepsilon_{S}}n_{tot}$. (S2-1)

To simplify Eq.(S2-1), we can employ a gradual approximation since channel length (along the y-axis) is long enough compared to film thickness (t_S_) along the x-axis [S2-1], as follows,

$\frac{\partial^{2}\phi(x,y)}{\partial x^{2}}>>\frac{\partial^{2}\phi(x,y)}{\partial y^{2}}$. (S2-2)

With Eq.(S2-1), Eq.(S2-2) is now simplified as a 1-dimensional equation,

$\frac{d^{2}\phi(x)}{dx^{2}}=\frac{q}{\varepsilon_{S}}n_{tot}$. (S2-3)

Besides this, E-field formula as a 1-D gradient of ϕ is given to be combined with Eq.(S2-3),

$E=-\frac{d\phi(x)}{dx}$. (S2-4)

Eqs.(S2-3) and (S2-4) yields the following integral form,

$\int_{0}^{E_{S}} EdE=\frac{q}{\varepsilon_{S}}\int_{0}^{\phi_{S}} n_{tot}d\phi$. (S2-5)

The boundary conditions are listed in the table shown below.

| x | ϕ(x) | E | Remarks |
| --- | --- | --- | --- |
| 0 | ϕ_S_ | E_S_ | Front interface |
| t_S_ | 0 | 0 | Back interface |

And the solution of Eq.(S2-5) is given as,

$E_{S}=\sqrt{\frac{2q}{\varepsilon_{S}}\int_{0}^{\phi_{S}} n_{tot}d\phi}$. (S2-6)

Here, n_tot_ is the sum of the following components: n_deep_, n_tail_, and n_free_.

The differential form is given as,

$n_{tot}=\frac{\varepsilon_{S}}{2q}\frac{dE_{S}^{2}}{d\phi_{S}}$. (S2-7)

Here, E_S_ is defined with Gauss’ law, as follows,

$E_{S}=C_{ox}(V_{GS}-V_{FB})$. (S2-8)

**Reference:**

[S2-1] S. M. Sze, *Physics of Semiconductor Devices*, 2nd ed. Hoboken, NJ: Wiley, 1981.

**█ S3. Flat-band voltage and its Approximation**

Since the flat-band voltage is fussy to be measured, we use an alternative one (V_R_) which is clear as a gate voltage at which drain current starts increasing. This is consistent with the following relations,

$C_{ox}(V_{GS}-V_{FB})=C_{ox}(V_{GS}-V_{R})+C_{ox}(V_{R}-V_{FB})$, (S3-1)

$C_{ox}(V_{GS}-V_{R})>>C_{ox}(V_{R}-V_{FB})$ $\Rightarrow C_{ox}(V_{GS}-V_{FB})\approx C_{ox}(V_{GS}-V_{R})$, (S3-2)

$V_{FB}\approx V_{R}$. (S3-3)

**█ S4. Transconductance before and after removal contact resistance effect (R_C_)**

Recalling Eq.(S1-8),

$I_{DS}=\mu_{b}Q_{free}\frac{W}{L}V_{DS}$, (S4-1)

the transconductance (g_m_) can be given as follows,

$g_{m}=\frac{dI_{DS}}{dV_{GS}}=\mu_{b}\frac{dQ_{free}}{dV_{GS}}\frac{W}{L}V_{DS}$. (S4-2)

In Eq.(S4-2), we assume that the only term as a function of V_GS_ is Q_free_. This implies that there is no effect of contact resistance (R_C_). However, in a real TFT, there is a non-negligible effect of R_C_. In order to consider this, we need to put R_C_ term into V_DS_ term as [S4-1,2,3],

$V_{DS}\to V_{DS}-R_{C}I_{DS}$. (S4-3)

Along with this, Eq.(S4-1) is rewritten as follows,

$I_{DS}=\mu_{b}Q_{free}\frac{W}{L}\left(V_{DS}-R_{C}I_{DS}\right)$. (S4-4)

Also, the closed form of Eq.(S4-4) is

$I_{DS}=\frac{\mu_{b}Q_{free}\frac{W}{L}V_{DS}}{1+\mu_{b}Q_{free}\frac{W}{L}R_{C}}$. (S4-5)

So, g’_m_ after removal of effect of R_C_ is given as,

$g'_{m}=\frac{g_{m}}{\left(1+\mu_{b}Q_{free}\frac{W}{L}R_{C}\right)^{2}}$. (S4-6)

Here, g_m_ is from Eq.(S4-2), and the main unknown Q_free_ can be extracted from,

$Q_{free}=\frac{I_{DS}}{\mu_{b}\frac{W}{L}\left(V_{DS}-R_{C}I_{DS}\right)}$. (S4-7)

**References:**

[S4-1] S. Lee *et al.,* *J. Display Tech*. **9**(11), 883 (2013).

[S4-2] S. M. Sze, *Physics of Semiconductor Devices*, 2nd ed. Hoboken, NJ: Wiley, 1981.

[S4-3] P. Servati et al., *IEEE Tran. Elec. Devi*. **50**(11), 2227 (2003).

**█ S5. Schematic Profiles of DOS for different PBS conditions**

The shape of peak in ∂^2^I_DS_/∂V_GS_^2^ appears to spread out more after applying the PBS for a longer t_PBS_. Indeed, the slope below V_T_ becomes less steep, suggesting a creation of deep defects [S5-1]. Since the peak envelope above V_T_ also becomes more moderate, as seen in Fig.5(d) in the manuscript, it can also be argued that tail states are also newly created only near E_T_. This situation has been schematically described in Fig.S5.1.


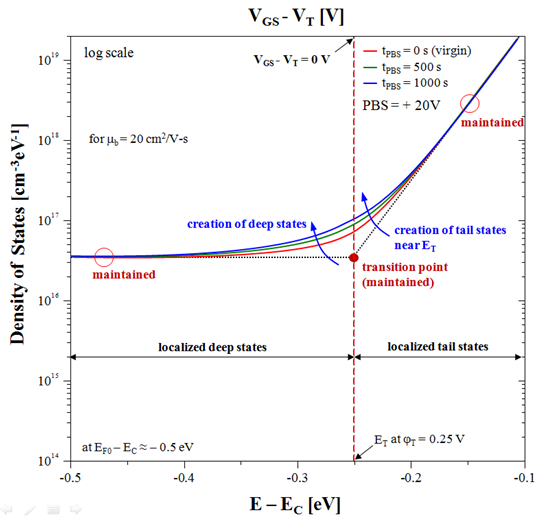


**Figure S5.1:** Schematic Profiles of DOS for different PBS conditions.

**Reference:**

[S5-1] M. J. Powell and S. C. Deane*, Phys. Rev. B* **48**, 10815 (1993).

**█ S6. Schematic Profiles of DOS in terms of the role of the gap states**

**Case 1) Role of N_deep_**


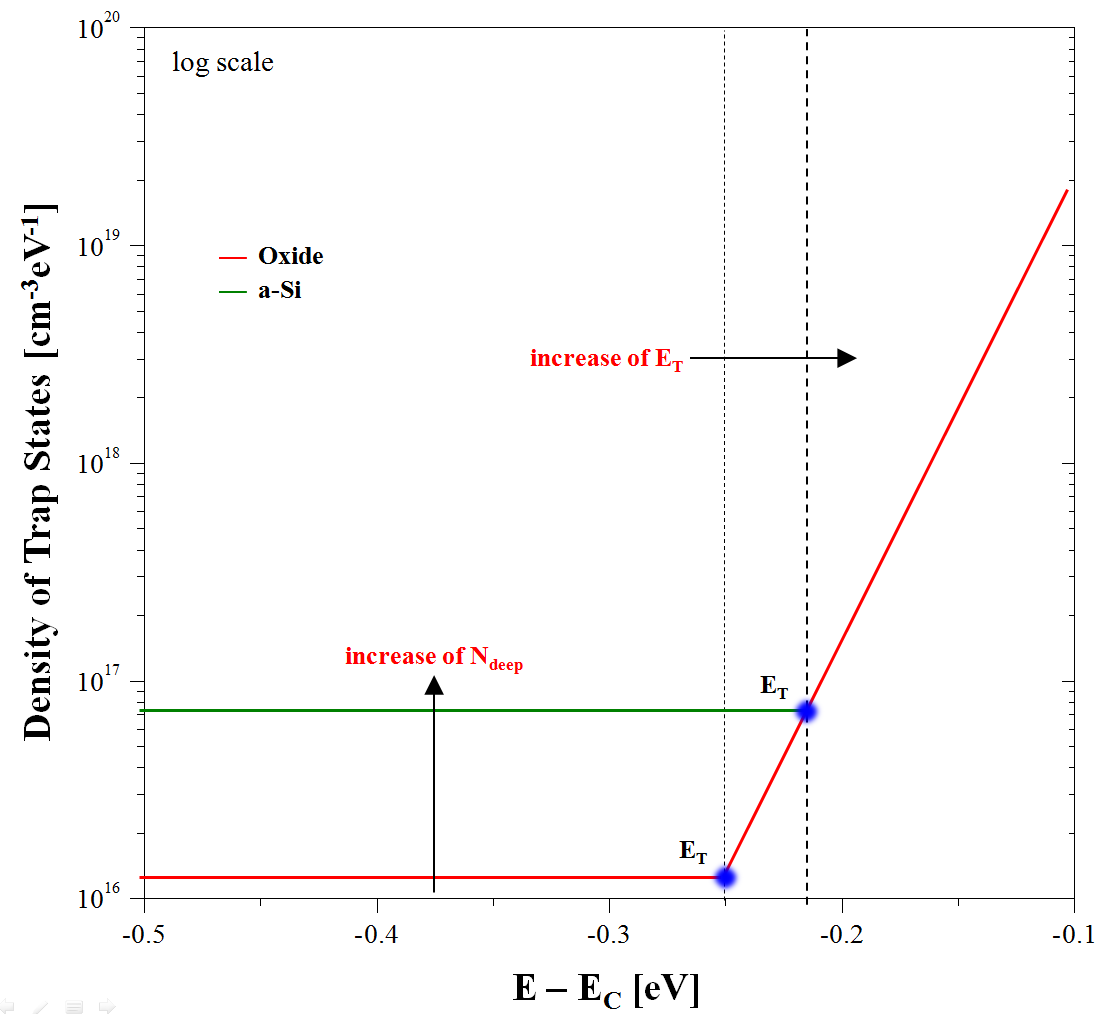


**Case 2) Role of N_tc_**


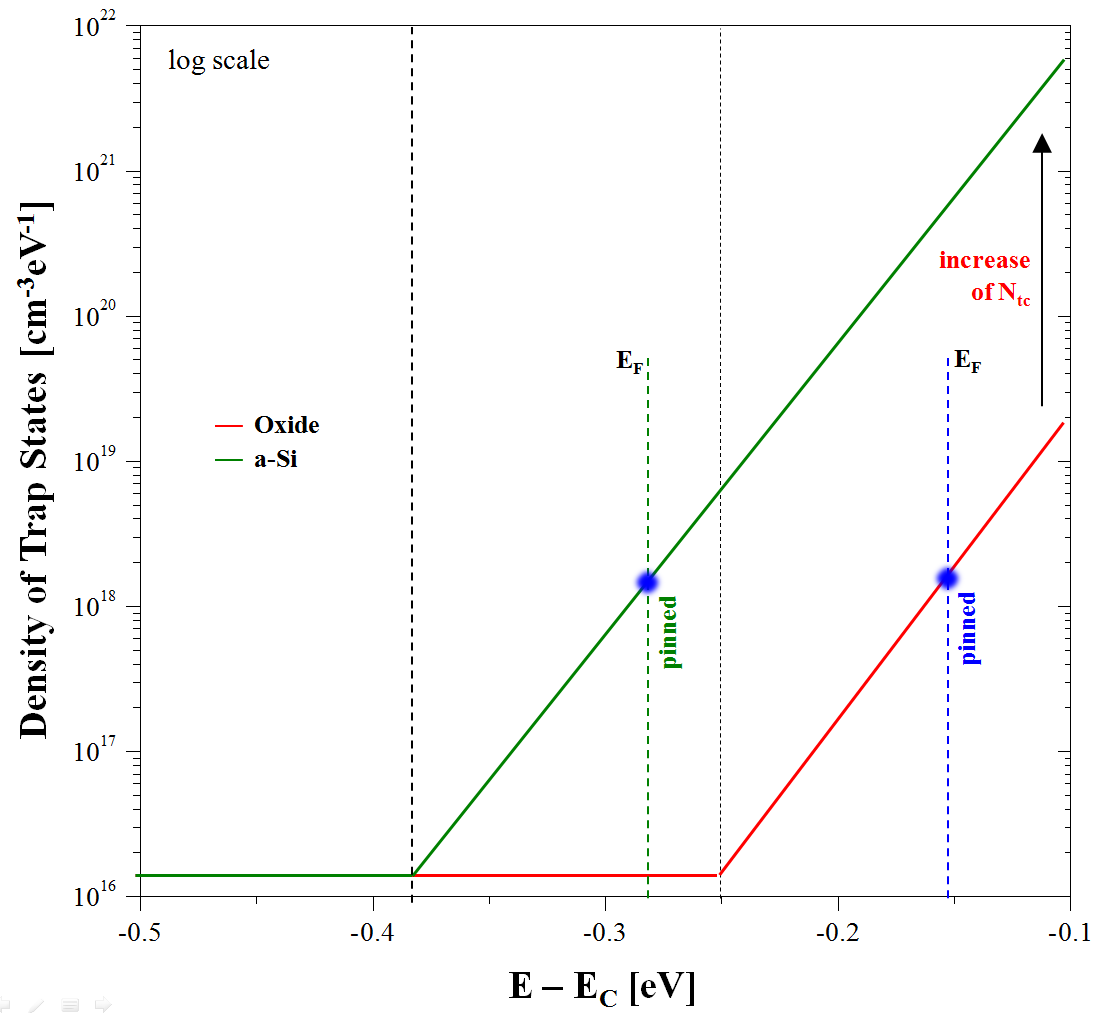


**Reference:**

[S6-1] R. Martins *et al.,* Journal of Applied Physics 101, 044505 (2007).
